# Assessing the role of undetected colonization and isolation precautions in reducing Methicillin-Resistant *Staphylococcus aureus *transmission in intensive care units

**DOI:** 10.1186/1471-2334-10-29

**Published:** 2010-02-16

**Authors:** Theodore Kypraios, Philip D O'Neill, Susan S Huang, Sheryl L Rifas-Shiman, Ben S Cooper

**Affiliations:** 1 School of Mathematical Sciences, University of Nottingham, Nottingham, UK; 2 Division of Infectious Diseases and Health Policy Research Institute, University of California Irvine School of Medicine, Irvine, California, USA; 3 Channing Laboratory, Brigham and Women's Hospital and Harvard Medical School, Boston, Massachusetts, USA; 4 Department of Ambulatory Care and Prevention, Harvard Medical School and Harvard Pilgrim Health Care, Boston, Massachusetts, USA; 5 Centre for Clinical Vaccinology and Tropical Medicine, Nuffield Department of Clinical Medicine, University of Oxford, Oxford, UK; 6 Faculty of Tropical Medicine, Mahidol University, Bangkok, Thailand

## Abstract

**Background:**

Screening and isolation are central components of hospital methicillin-resistant *Staphylococcus aureus *(MRSA) control policies. Their prevention of patient-to-patient spread depends on minimizing undetected and unisolated MRSA-positive patient days. Estimating these MRSA-positive patient days and the reduction in transmission due to isolation presents a major methodological challenge, but is essential for assessing both the value of existing control policies and the potential benefit of new rapid MRSA detection technologies. Recent methodological developments have made it possible to estimate these quantities using routine surveillance data.

**Methods:**

Colonization data from admission and weekly nares cultures were collected from eight single-bed adult intensive care units (ICUs) over 17 months. Detected MRSA-positive patients were isolated using single rooms and barrier precautions. Data were analyzed using stochastic transmission models and model fitting was performed within a Bayesian framework using a Markov chain Monte Carlo algorithm, imputing unobserved MRSA carriage events.

**Results:**

Models estimated the mean percent of colonized-patient-days attributed to undetected carriers as 14.1% (95% CI (11.7, 16.5)) averaged across ICUs. The percent of colonized-patient-days attributed to patients awaiting results averaged 7.8% (6.2, 9.2). Overall, the ratio of estimated transmission rates from unisolated MRSA-positive patients and those under barrier precautions was 1.34 (0.45, 3.97), but varied widely across ICUs.

**Conclusions:**

Screening consistently detected >80% of colonized-patient-days. Estimates of the effectiveness of barrier precautions showed considerable uncertainty, but in all units except burns/general surgery and one cardiac surgery ICU, the best estimates were consistent with reductions in transmission associated with barrier precautions.

## Background

Patient isolation measures (such as barrier precautions or physical isolation of patients in single rooms or cohorts) and screening to detect MRSA colonization are core components of almost all policies for preventing the nosocomial transmission of methicillin-resistant *Staphylococcus aureus *(MRSA). However, there are few well-designed intervention studies considering screening and isolation precautions in the absence of other containment measures, and little is known about the extent to which they reduce transmission. Obtaining estimates of any such reduction is important for both quantifying the value of existing practices and the potential benefit of new rapid screening technologies.

Screening and isolation measures have two complementary aims: to minimize the number of days MRSA-positive patients are left unisolated; and to minimize MRSA transmission from isolated patients. In this paper we consider the extent to which we can quantify the success of control programs in achieving both aims using routine surveillance data. This presents a number of challenges. First, the number of unisolated MRSA patient days usually cannot be directly observed using surveillance data, because in most settings those who are known to be MRSA-positive are immediately isolated. Moreover, screening cultures are typically taken no more than weekly. This, combined with less than perfect swab sensitivity, makes it impossible to directly observe the total number of colonized patient days, the number of transmission events and the precise times at which these events occur. Furthermore, estimating the effectiveness of isolation measures in reducing transmission requires quantifying the relative transmission rates of both known and isolated and unobserved and unisolated MRSA positive patients. This cannot be done without estimates of the number of days MRSA-positive patients are left unisolated. Together, these obstacles prevent direct estimates of the efficacy of patient isolation in reducing transmission using routine surveillance data.

Here we show that data augmentation methods allow these problems to be overcome by treating the unobserved times at which patients acquire MRSA as unknown parameters to be estimated. By analysing such surveillance data using stochastic transmission models and making use of data augmentation techniques it is possible to simultaneously estimate the number of undetected MRSA positive patient days and the efficacy of isolation measures in reducing transmission using routinely collected MRSA surveillance data.

## Methods

### Data Collection

We collected detailed census and microbiologic data from eight adult 10-bed intensive care units (ICUs) in a tertiary academic medical center in Boston, Massachusetts where routine admission and weekly bilateral nares screening for MRSA was occurring with high compliance (90%). Types of ICUs included medical, cardiac, general surgery (2), neurosurgery, thoracic surgery, and cardiac surgery (2). One of the two general surgery wards also received burn and trauma patients. Dates of MRSA-positive clinical cultures (all sources), as well as positive and negative MRSA nares screening cultures were collected during a 17-month period from September 2003 through January 2005. Negative clinical cultures were not assessed. All specimens were processed by routine bacterial culturing techniques. This study was approved by the Institutional Review Board at Brigham and Women's Hospital with a waiver of informed consent.

Newly-identified and previously known MRSA-positive patients were placed under contact precautions consisting of gown and glove use as well as use of single rooms (all ICU rooms were single occupant). Once identified as MRSA-positive, contact precautions were applied on that admission and all subsequent admissions. No nurse cohorting was utilized. Dates of each ICU admission and discharge were obtained, along with the date on which contact precautions were initially applied (for MRSA or other highly antibiotic resistant pathogens). The first institutional date of a MRSA-positive culture was also recorded, even if it preceded the study period.

### Data analysis

#### Stochastic model

Our baseline analysis used a previously described dynamic stochastic single-ward transmission model to analyse the data [1]. At any point in time, each patient is assumed by this model to be in one of two states, colonized (defined here as the presence of MRSA at any body site, regardless of symptoms) or uncolonized. Each patient entering the ward has a probability ϕ of being already colonized, unless they are already known to be so (i.e. having already had a previous positive test).

During a stay on the ward, an initially uncolonized patient is assumed to have a risk of becoming colonized that can be described by a Poisson process: this means that the probability of a patient becoming colonized between time *t *and time *t*+*τ *can be approximated by *λ (t) τ *(where *τ *is a small interval of time, the approximation becoming exact as *τ *approaches zero). Consequently, a patient's chances of becoming colonized increase with length of stay on the ward. The rate λ *(t) *can vary through time; since we are interested in comparing different potential sources of colonization we assume that λ *(t) = β_0 _+ C(t)β_1 _+ I(t)β_2_*. Here, *β*_0 _is the rate of background transmission, *β*_1 _is the rate of transmission due to colonized but nonisolated patients, *β*_2 _is the rate of transmission due to colonized and isolated patients, and *C(t) *and *I(t) *are the numbers of nonisolated and isolated colonized patients at time *t*, respectively. We thus assume that the "colonization pressure" on an uncolonized individual increases linearly with the number of colonized patients who are isolated and nonisolated. Although it is common practice to use a scaling such as *β*_1_/*N *instead of *β*_1_, with *N *the typical number of patients on the ward, this did not seem necessary since there was very little variation in ward occupancy both between wards and between different times during the study period. The inclusion of the background rate *β*_0 _models the assumption of a constant risk of colonization irrespective of the presence of colonized patients. Such background transmission could, for example, result from environmental contamination or contact with staff carriers of MRSA [2-4]. Note that the model takes no explicit account of HCW compliance with barrier precautions.

Once a patient is colonized, we assumed that he or she remains so for a three month period. Thus, a patient colonized on discharge is assumed to still be colonized if readmitted within three months. This assumption was not critical: the results presented below were virtually unchanged when different periods of colonization were assumed (e.g. 6 months, 9 months). We assumed that the nares culture had a specificity of 100% and a sensitivity of *p *× 100%, where *p *(the sensitivity of a nasal swab for detecting MRSA carriage at any site) was estimated from the data.

#### Statistical inference

The above model has five main parameters, namely ϕ, *p, β*_0_,*β*_1_, and *β*_2_. We wish to estimate these from the data, which consist of admission times, discharge times, and the times and outcomes of tests. Since our approach involves estimating unseen colonization times, we can also estimate quantities such as the duration of carriage prior to a positive test result. Assessment of the effectiveness of isolation is performed by comparing estimates of *β*_1 _and *β*_2_: evidence that *β*_1 _> *β*_2_would support the hypothesis that isolation is effective in reducing transmission. Specifically, we estimate P(*β*_1 _> *β*_2_) and the ratio *β*_1_/*β*_2 _. Estimation was carried out within a Bayesian statistical framework using Markov chain Monte Carlo (MCMC) methods [5].

The five model parameters were assigned uninformative and independent prior distributions: Uniform(0,1) distributions for ϕ and *p*; and exponential distributions with rate 10^-6 ^for each of the *β *parameters. Since the likelihood of the observed data (dates and outcomes) given the model parameters is computationally intractable, we used a data augmentation method in which the unobserved colonization times were included as additional parameters. This yields an augmented posterior density that is known up to proportionality, which we explored using an MCMC algorithm. Within the algorithm, infection rate parameters were updated using Metropolis-Hastings steps, while both ϕ and *p *were updated using Gibbs steps [6].

Our methods enable us to estimate the percent of patient days that are attributed to patients who are colonized but not yet detected (*p*_*hidden*_), and the percent of patient days attributed to patients who are colonized and have been tested, but who are awaiting test results (*p*_*wait*_). Note that *p*_*hidden *_> *p*_*wait *_since all "waiting" patients are also "hidden".

Some of the parameter estimates were combined using a random effects model with inverse variance weights [[7], Sec. 5.2] to derive pooled estimates and corresponding standard errors. These computations were carried out using the rmeta package for R http://www.r-project.org. All other analysis, such as the implementation of the MCMC algorithms, was performed using programs we wrote in C.

#### Sensitivity analysis

We refer to the model described above as the *full model*. To assess the impact of our assumptions, we repeated the analyses using two alternative models. In the first (the *no-background model*), *β*_0 _is assumed to be zero with high probability (specifically, *β*_0 _was assigned an exponential prior distribution with mean 10^-4 ^so P(*β*_0 _< 0.001) > 0.99). This corresponds to the assumption that virtually all of the MRSA acquisitions result from patient-to-patient transmission (much of which is presumably mediated by transiently colonized healthcare workers, though this is not explicitly modeled). Background transmission unrelated to colonization pressure, e.g. environmental contamination, is assumed to be of negligible importance. In the second (the *non-linear model*), the assumption of linearly increasing colonization pressure is relaxed, and it is instead assumed that colonization pressure due to both isolated and nonisolated individuals remains constant provided at least one colonized individual is present. Although biologically less plausible than the full model, this model represents an extreme case and by fitting it we obtain insights into the impact of the assumptions underlying the full model. For both the non-linear and no-background models, assessment of the effect of isolation is measured via comparison of the two rates *β*_1 _and *β*_2 _as described above.

#### Test sensitivity

We consider two definitions of swab sensitivity: swab sensitivity in detecting colonization of the nares, and swab sensitivity for detecting colonization at any body site. The former is calculated from a subset of the data (without making use of the model) by comparing subsequent swabs in patients who have a first positive swab. Precisely, the sensitivity is estimated by the ratio TP/(TP+FN) using serial nares cultures alone, where TP and FN denote total numbers of true positive and false negative tests on a ward excluding the first positive swab from each patient. Since patients, once colonized, are assumed to remain so throughout their ICU stay, TP is simply the number of positive swabs excluding the first for each patient episode, and FN the number of negative swabs that follow an earlier positive for the same patient. The second reported sensitivity is the parameter *p *estimated from the model making use of cultures from all sites (this is necessarily lower than the sensitivity for detecting nares carriage).

## Results

Descriptive characteristics of the individual ICUs are shown in tables 1 and 2. The median length of stay was two days in all but the two General Surgical wards, in which it was one day. A total of 4,977 MRSA positive cultures were collected from the study population. Thirty three percent of these cultures were nares screening cultures.

**Table 1:** Summary statistics for the study data

Ward^a^	Number of patients	Length of stay Mean(SD)	Percent in contact precaution^b^	Number of swab tests per person Mean (SD)	Number of MRSA+ swab tests per person Mean (SD)	Number of swab tests taken after first positive^c^
M1	1293	3.4 (4.7)	11.4	1.4 (0.8)	0.13 (0.5)	73
M2	1018	4.4 (6.4)	19.1	1.5 (0.9)	0.20 (0.61)	45
GS1	1227	3.4 (5.2)	12.4	1.3 (0.7)	0.14 (0.5)	63
GS2	1030	4.0 (8.3)	10.7	1.4 (0.9)	0.14 (0.5)	99
SS1	706	5.8 (11.4)	12.5	1.3 (0.9)	0.10 (0.5)	100
SS2	888	4.8 (9.7)	7.5	1.4 (1.1)	0.11 (0.6)	111
SS3	1097	3.8 (6.4)	6.0	1.2 (0.7)	0.05 (0.3)	51
SS4	1263	3.6 (5.2)	5.1	1.4 (0.8)	0.07 (0.3)	42

^a ^Here M denotes Medical or Cardiac ICU, GS denotes General Surgical ICU, including trauma and burn patients, SS denotes Specialty Surgical ICU.

^b ^Includes isolation indicators other than MRSA.

^c ^The total number of swab tests taken after the first MRSA-positive test.

**Table 2:** Observed prevalence and incidence for the study data

Ward^a^	Monthly MRSA prevalence^b^ Mean (SD)	Monthly MRSA admission prevalence^c^ Mean (SD)	Monthly MRSA incidence density (new cases per 1,000 patient days) Mean (SD)
M1	16.9 (4.0)	12.6 (3.0)	8.8 (8.1)
M2	23.5 (4.7)	20.6 (3.3)	5.8 (5.8)
GS1	20.5 (5.8)	15.4 (4.2)	9.3 (8.3)
GS2	19.5 (6.4)	10.9 (4.1)	18.2 (9.7)
SS1	21.1 (8.7)	12.9 (7.5)	9.8 (10.1)
SS2	13.8 (4.4)	7.3 (2.8)	6.8 (4.9)
SS3	10.7 (3.9)	6.3 (3.6)	9.4 (7.9)
SS4	9.2 (3.3)	4.5 (3.1)	9.1 (6.1)

^a ^Here M denotes Medical or Cardiac ICU, GS denotes General Surgical ICU, including trauma and burn patients, SS denotes Specialty Surgical ICU.

^b ^The number of ICU patients ever known to be MRSA-positive before or during that month, divided by the total number of ICU patients that month.

^c ^The number of patients ever known to be MRSA positive before or within two calendar days of admission, divided by the total number of monthly admissions.

**Figure 1:**
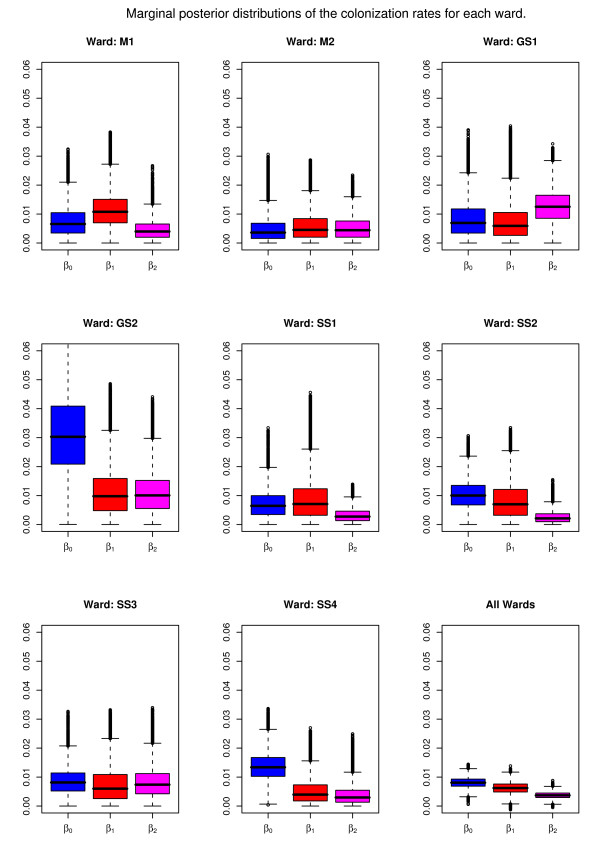
**Marginal posterior distributions of the colonization rates for each ward**. Boxplots of the marginal posterior distributions are shown which contain (the smallest observation, lower quartile (Q1), median (Q2), upper quartile (Q3), and largest observation (sample maximum) as well as any outliers. The whiskers extend from the edges of the box to the most extreme data point which is no more than 1.5 times the interquartile range away from the box.

Estimates for *β*_1 _were fairly consistent across the wards while *β*_0 _and *β*_2 _exhibited more variation (figure 1). General surgery wards were distinguished by the fact that the estimated transmission rate with barrier precautions (β_2_) was found to be higher than that without (β_1_). Posterior distributions of the three rates were found to be negatively correlated. This was expected since an increase in one rate must be accounted for by a decrease in another. The background rate *β*_0 _had considerable correlation with the other two rates (range -0.23 to -0.58), while *β*_1 _and *β*_2 _were far less strongly correlated (range -0.05 to -0.30).

### The impact of undetected colonized patients on transmission

Approximately 15% of colonized patient days (CPDs) were due to undetected patients, this being made up of around 10% due to patients awaiting test results, and about 5% due to patients who had not been tested (table 3). The fraction of CPD attributed to undetected patients was not statistically associated with the mean monthly overall prevalence or admission prevalence of MRSA in each ICU (Pearson's correlation coefficient -0.58 and -0.53, p-values 0.13, 0.17, respectively.)

**Table 3:** Assessment of undetected colonization

Ward	Full model		No-background		Non-linear	
	
	*p*_*hidden*_	*p*_*wait*_	*p*_*hidden*_	*p*_*wait*_	*p*_*hidden*_	*p*_*wait*_
M1	16.5 (14.9, 18.2)	11.5 (10.6, 12.4)	16.6 (14.9, 18.2)	11.4 (10.5, 12.2)	16.4 (14.8, 18.1)	11.5% (10.6, 12.3)
M2	10.5 (8.3, 13.2)	6.7 (5.6, 7.9)	10.8 (8.4, 13.7)	6.9 (5.8, 8.3)	10.4 (8.1, 13.3)	6.7 (5.5, 8.0)
GS1	13.8 (12.2, 15.5)	8.3 (7.6, 8.9)	13.9 (12.3, 15.6)	8.3 (7.6, 8.9)	13.9 (12.2, 15.5)	8.3 (7.5, 8.9)
GS2	17.3 (15.1, 19.8)	7.4 (6.6, 8.2)	17.3 (15.1, 19.6)	7.3 (6.6, 8.0)	17.4 (15.1, 19.7)	7.5 (6.7, 8.2)
SS1	9.6 (8.1, 11.2)	4.7 (4.4, 4.9)	9.9 (8.4, 11.6)	4.7 (4.4, 4.9)	9.6 (8.1, 11.2)	4.7 (4.4, 4.9)
SS2	10.7 (9.0, 12.7)	5.7 (5.4, 6.1)	10.9 (9.1, 13.0)	5.8 (5.4, 6.1)	10.7 (8.9, 12.8)	5.7 (5.4, 6.1)
SS3	15.6 (13.0, 18.4)	7.9 (6.9, 8.7)	16.2 (13.5, 18.9)	7.8 (6.9, 8.6)	15.5 (12.7, 18.3)	7.9 (6.9, 8.7)
SS4	19.8 (16.2, 23.3)	10.1 (8.7, 11.4)	20.7 (17.1, 24.3)	9.7 (8.4, 10.9)	19.8 (16.5, 23.3)	10.1 (8.6, 11.3)

Note: Model estimates (95% credible intervals) of the mean percent of colonized-patient-days attributed to undetected colonized patients (*p*_*hidden*_), and the mean percent of colonized-patient-days attributed to colonized patients who had been swabbed but were waiting for results (*p*_*wait*_).

### Is isolation effective? Comparison of *β*_1 _and *β*_2_

In five of the eight wards there was consistent, though weak, evidence that *β*_1 _is larger than *β*_2 _indicating that isolation is associated with reduced transmissibility (table 4). The probability of an isolation benefit varied widely across the units, e.g. 27-82% under the full model.

**Table 4:** Assessment of isolation efficacy

Ward	Full model		No-background		Non-linear	
	
	P(*β*_1 _> *β*_2_)	Median (*β*_1_/*β*_2_)	P(*β*_1 _> *β*_2_)	Median (*β*_1_/*β*_2_)	P(*β*_1 _> *β*_2_)	Median (*β*_1_/*β*_2_)
M1	0.82	2.7	0.83	2.4	0.75	2.3
M2	0.51	1.0	0.54	1.1	0.45	0.8
GS1	0.27	0.5	0.15	0.3	0.36	0.6
GS2	0.50	1.0	0.58	1.2	0.62	1.5
SS1	0.73	2.7	0.57	1.3	0.53	1.1
SS2	0.79	3.3	0.79	2.0	0.71	2.0
SS3	0.44	0.8	0.60	1.3	0.67	2.0
SS4	0.58	1.3	0.70	2.3	0.59	1.4

Note. P(*β*_1 _> *β*_2_), the probability that *β*_1 _is larger than *β*_2_, measures the strength of the evidence that isolation was associated with reduced transmission (a value of 1 would indicate certainty), and the ratio of *β*_1 _to *β*_2 _measures the relative infectivity of unisolated compared to unisolated patients.

### Pooled estimate of the effectiveness of isolation

**Figure 2:**
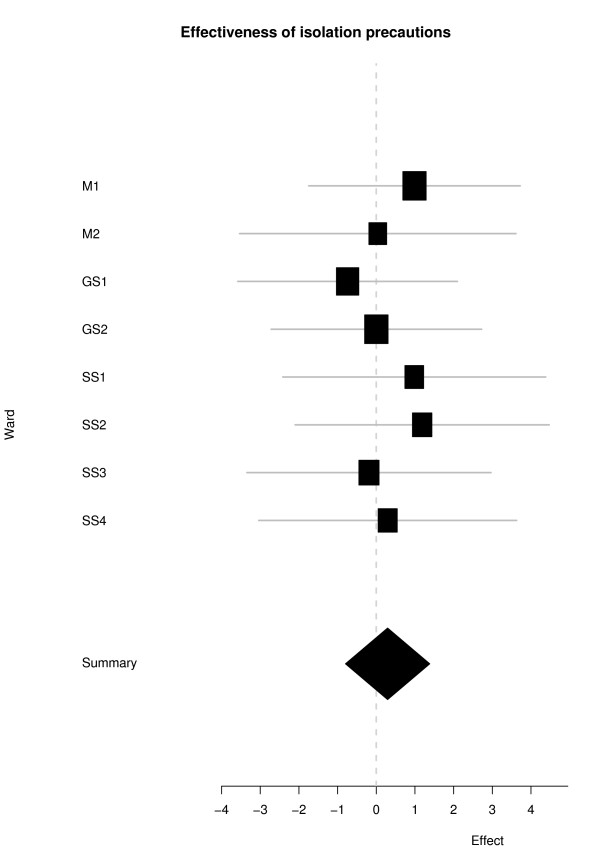
**Effectiveness of isolation precautions**. Forest plot showing individual and pooled estimate of ln(β_1_/β_2_) for each ward. Horizontal lines are 95% CIs and the size of each square is proportional to weight in the meta-analysis. The end points of the summary diamond indicate 95% CI.

Although our analysis treats each ward individually, it is nevertheless of interest to consider a pooled estimate of isolation efficacy. Such an estimate of (*β*_1_/*β*_2_) across all wards has mean (95% credible interval) 1.34 (0.45, 3.97) which indicates some evidence to support the efficacy of isolation, but a relatively high degree of uncertainty. This estimate can be decomposed into contributions from medical, specialty surgical and general surgical wards, yielding 1.89 (0.21 16.60), 1.73 (0.33 8.94) and 0.7 (0.1, 5.0) respectively. Variation across all wards is illustrated in Figure 2, which shows a pooled estimate of log(*β*_1_/*β*_2_) for the full modeland the corresponding standard deviation for each ward. In addition, the value of the I^2 ^statistic, which describes the percentage of variation across wards that is due to heterogeneity, was equal to zero for the aforementioned pooled estimates.

### Test sensitivity

We report the two estimates of swab sensitivity defined in the methods section (table 5). Overall, the sensitivity of detecting colonization at any body site was estimated from the model to be 60% (95% 55%-63%) while the sensitivity of the nares test was estimated directly from the data to be 85%.

**Table 5:** Two measures of nares test sensitivity based upon serial nares cultures

Ward	Sensitivity of Detecting Nares Colonization (%)^a^	Sensitivity of detecting colonization at any body site (%): Median (95% CI)^b^
M1	92	64 (56, 71)
M2	84	56 (47, 65)
GS1	82	61 (54, 69)
GS2	88	50 (42, 57)
SS1	89	62 (54, 68)
SS2	89	68 (60, 76)
SS3	85	55 (45, 64)
SS4	72	54 (44, 65)
Overall	85	59 (55, 63)

^a ^Given by the ratio TP/(TP+FN) where TP is the number of positive swabs excluding the first for each patient episode and FN the number of negative swabs that follow an earlier positive for the same patient.

^b ^The parameter *p *estimated from the model making use of cultures from all sites.

### Percentage of new admissions already colonized

Each patient admitted to a ward during the study period may be known to be colonized due to a previous positive test. If not, colonization status is unknown, since the patient may be either new to the study, or else previously admitted but with no positive test result. The parameter ϕ in our model represents the proportion of such patients who are colonized on admission. Note that ϕ should not be interpreted as a measure of admission prevalence, since it excludes known positives.

Estimates of ϕ were found to be similar to the percentage of patients admitted with a known previous positive test on all wards except M2, with the lowest values tending to occur in the specialist surgery wards (table 6). Estimates from the full model and no-background model were similar, as were estimates from the non-linear model (not shown).

**Table 6:** Percentage of patients who are colonized on admission to a ward

Ward No	**Percent having a previous positive test **^a^	**Estimated percent of MRSA carriers among admissions with unknown status**^b^**(ϕ × 100%): Median (95% CI)**	
		
		Full Model	No Background
M1	8.4	9.4 (7.3, 12)	9.6 (7.5, 12.2)
M2	7.7	14.1 (10.3, 18.6)	14.4 (10.7, 19)
GS1	8.1	10.4 (7.8, 13.4)	10.7 (8.1, 13.9)
GS2	5.7	8.3 (5.4, 12.1)	9.0 (6, 13)
SS1	8.2	8.7 (5.9, 12.2)	9.2 (6.4, 12.7)
SS2	4.6	5.7 (3.8, 8.2)	6.1 (4.2, 8.6)
SS3	4.2	3.4 (2.1, 5.3)	3.8 (2.4, 5.8)
SS4	3.5	4.6 (2.9, 6.9)	5.9 (4.0, 8.4)

^a ^Patients known to be colonized due to having had a previous positive test during the study period (column 2).

^b ^The percentage of patients with unknown status (due to being newly-admitted or with no previous positive test) who are estimated to be colonized on admission by the model.

## Discussion

We have shown that a data-augmentation approach allows us to estimate the extent of undetected MRSA carriage and the effectiveness of barrier precautions (gown and gloves) in preventing transmission using only routinely collected high-compliance admission and weekly MRSA screening cultures. Such estimates would have been difficult or impossible with more conventional approaches and would have required assuming rather than estimating the unobserved carriage data.

Our results have shown that admission and weekly nares screening with conventional culture methods are likely to have detected at least 80% of MRSA colonized patient days in the ICUs studied, despite 48-hour culture turn-around times, and sensitivities to detect MRSA carriage of only 85% for detecting nares carriage and 59% for carriage at any body site. The explanation for this high detection of MRSA patient days despite a low sensitivity for non-nares sites is that clinical cultures of other sites are routinely being performed for medical reasons. Thus, nares screening in conjunction with usual routine culturing performed for medical reasons captures a large majority of positive MRSA carriers. While generalizability to non-ICU wards remains unclear, this level of detection in ICUs is reassuring since risk of MRSA infection is highest in these settings.

We estimate that approximately 10% of potential contact precaution days are missed due to delays in obtaining test results. If contact precautions cause a 28.1% reduction in the transmission rate (i.e. 1 - (*β*_2_/*β*_1_), using the pooled estimate from all the wards), then an additional 5% reduction in total transmission rates would be gained with instantaneous swab results, suggesting a limit to the potential benefit in using rapid screening technologies. However, the potential benefit of rapid test results would increase in line with the effectiveness of isolation measures.

While our best estimate is that gown and glove barrier precautions produce a 28% reduction in MRSA transmission, we recognize that these estimates have considerable uncertainty despite including over 11 ward-years of data and there is a substantial chance that the effect is both much lower and much larger. Thus we estimate that there is 30% chance that the reduction in transmission is above 44% and a 30% chance that isolation actually increases transmission.

This uncertainty may indicate that many more years of data are required, and/or may be explained by the fact that data from different types of ICUs were pooled. However the Woolf's test for heterogeneity did not reach significance at a 5% level (p = 0.982), indicating that these differences are consistent with chance. Moreover since comparisons between ward-types were not planned they may be of value for generating, but not testing hypotheses.

Recognizing these caveats, it is plausible that barrier precautions have differential effectiveness in different types of ICUs. The estimates found in this study suggest that medical ICUs may derive the most benefit from barrier precautions. This is important since the MRSA prevalence in medical ICUs is often higher than in other ICU types. Interestingly, our results suggest that no benefit was derived in general surgery ICUs serving burn and trauma patients. It is possible that barrier precautions may be less effective when the burden of MRSA is profuse, such as with surgical wounds or burns. Such an interpretation is supported by the fact that the rate of background transmission (*β*_0_) is substantially higher on these units. This is also supported by other work in these units suggesting that contamination with MRSA is higher in surgical versus medical ICUs [8]. Another possible explanation for the lack of benefit seen in select wards is low compliance with contact precautions, although this was not assessed. Finally, if barrier precautions provide a false sense of security and lead to poorer hand hygiene, then transmission might effectively worsen with this practice.

It is also possible that the differing effectiveness of barrier precautions among ICUs could be due to unmeasured effects. Specifically, we did not measure or account for variations in host risk factors for acquisition (comorbidities, severity of illness, wounds, devices), nor did we measure the effect of other ongoing infection control interventions such as hand hygiene campaigns. We do note that infection control interventions were not differentially applied to certain ICUs during this time, but levels of compliance are not known. In the absence of reliable data about these factors, none were considered in our analysis.

Our results may underestimate the full effect of barrier precautions since all ICU rooms were single occupancy; thus, we could not assess the impact of this component. In addition, studies have suggested that compliance with barrier precautions is around 70% [9,10]. Our estimates represent estimates of "effectiveness" (under routine operation) rather than a theoretical efficacy that would apply only under perfect compliance. Thus, while our estimates can be considered conservative, we believe they are the most practically relevant outcome measures. More precise estimates would require more prolonged surveillance data, data with more frequent swabbing intervals, and/or extensive typing information to help identify transmission routes.

The model-based approach that we have adopted for data analysis has several advantages over standard statistical methods, yielding more powerful analyses based on more realistic assumptions. In particular, it accounts for unobserved but important events (e.g. the time at which a patient becomes colonized), and attempts to estimate these events and their uncertainty. It also is based upon biologically meaningful assumptions regarding transmission, addressing both environmental sources and patient-to-patient transfer.

In summary, we have used a stochastic model to estimate the importance of undetected MRSA carriage and the impact of gown and glove precautions on MRSA transmission from 11-ICU years of active MRSA surveillance data. Such isolation precautions are the foundation of infection control guidelines for MRSA control. Nevertheless, there are concerns that isolation techniques may reduce the quality of patient care and incur risks related to inattention [11-13]. Thus, quantifying the beneficial effects and understanding the types of hospital wards in which such measures are likely to be insufficient is a critical part of weighing the need for additional or alternative measures. This work shows that even with conventional MRSA detection technology and weekly screening, a very large proportion of the total patient MRSA reservoir can be detected. It also suggests that barrier precautions may afford substantial benefit in medical and possibly specialty surgical ICUs, but that barriers to its effectiveness may need further study. The large uncertainty in effectiveness estimates, however, illustrate the limitations of routine surveillance data and highlight the need for well-designed prospective intervention studies to evaluate such interventions with greater precision.

## Conclusions

In a study of 8 ICUs performing admission and weekly nares surveillance, the average percentage of colonized-patient-days attributed to undetected carriers was 14%, while the percentage of colonized-patient-days attributed to patients awaiting test results was 8%. This suggests that nares surveillance identifies a large majority of carriers and that pcr testing may confer only a small benefit over routine culture. Estimates of the effectiveness of barrier precautions showed an overall benefit of 25%, but this benefit varied widely across different types of ICUs.

## Competing interests

The authors declare that they have no competing interests.

## Authors' contributions

TK coded and implemented the statistical analysis. BSC and PDO conceived the modeling and statistical framework, and drafted the manuscript. SSH contributed content expertise, oversaw dataset preparation for modeling, and helped draft the manuscript. SSR-S contributed to the data cleaning and preparation of the dataset for use in the models. All authors have read and approved the final manuscript.

## Pre-publication history

The pre-publication history for this paper can be accessed here:

http://www.biomedcentral.com/1471-2334/10/29/prepub
